# Determinants of work capacity (predicted VO_2max_) in non-pregnant women of reproductive age living in rural India

**DOI:** 10.1186/s12889-021-10785-x

**Published:** 2021-04-15

**Authors:** Loretta DiPietro, Jeffrey Bingenheimer, Sameera A. Talegawkar, Erica Sedlander, Hagere Yilma, Pratima Pradhan, Rajiv Rimal

**Affiliations:** 1 Departments of Exercise & Nutrition Sciences, Milken Institute School of Public Health, The George Washington University, Washington, DC, USA; 2 Departments of Prevention & Community Health, Milken Institute School of Public Health, The George Washington University, Washington, DC, USA; 3 DCOR Consulting, Bhubaneswar, Odisha India; 4 Department of Health, Behavior & Society, Bloomberg School of Public Health, Johns Hopkins University, Baltimore, MD USA

**Keywords:** Fitness, Human capital, Productivity, Women’s health

## Abstract

**Background:**

The negative impact of anemia on work capacity has been studied extensively in male and female workers; however, the simultaneous contributions of confounding variables such as physical activity, as well as other behavioral and sociodemographic characteristics have not been considered. The purpose of this study was to examine cross-sectionally the multivariable correlates of work capacity in non-pregnant women (*n* = 330) living in rural India.

**Methods:**

The Reduction in Anemia through Normative Innovations (RANI) Project is a norms-based, clustered randomized controlled trial to reduce anemia among women (15–49 years) living in Odisha, India between 2018 and 2021. For the larger trial, 89 clusters of villages were randomized into treatment and control groups on a 1:1 basis. Women (2055/group) living in 15 selected clusters (40–41 villages) were then randomly selected for data collection. The sampling design also randomly-generated a subset (*n* = 375) of non-pregnant participants who performed a modified Queen’s College Step Test (QCST) and who wore an activity monitor for 3 days. Predicted work capacity (VO_2max_) was determined using the QCST. Levels (h/day) of daily reclining, sitting, standing, walking (steps/day), and energy expenditure (MET∙h/day) were determined using an ActivPAL accelerometer. Hemoglobin concentrations (g/dL) were determined using a HemoCue photometer. Predetermined hierarchical (non-multilevel) regression models tested the independent associations between the primary study variables of interest (physical activity, hemoglobin concentrations) and predicted VO_2max_, while adjusting for age, body mass index (BMI: kg/m^2^), education, parity, and dietary diversity score.

**Results:**

Approximately 61% of the participants had anemia (Hb < 12 g/dL). Age^2^ (β = − 0.01; 95% CI: − 0.01, 0.00), BMI (β = − 0.19; 95% CI:-0.28, − 0.09), educational attainment (β = − 1.35; 95% CI: − 2.34, − 0.36), and MET∙h/day (β = 0.19; 95% CI: 0.00, 0.38) were significant and independent determinants of work capacity. Hemoglobin concentration was marginally associated with work capacity in the presence of the other covariables (β = 0.22; 95% CI:-0.02, 0.47).

**Conclusions:**

Our data indicate that factors other than anemia are important correlates of work capacity and should be considered when promoting the health and economic capacity of rural Indian women.

**Trial registration:**

Clinical Trial Registry- India (CTRI) http://ctri.nic.in/Clinicaltrials/pmaindet2.php?trialid=26285&EncHid=&userName=CTRI/2018/10/016186 on 29 October 2018.

**Supplementary Information:**

The online version contains supplementary material available at 10.1186/s12889-021-10785-x.

## Background

Human survival is dependent upon the capacity to perform the work necessary to ensure adequate food, shelter, and clothing [1]. The current reliance on modern technology in most developed countries has resulted in a steady decline in daily work-related energy expenditure [2]. This is not the case in still-developing societies, however, in which survival remains dependent on human power and prolonged physical work. In India, approximately 55% of women engage in agricultural labor, with the majority of them being of reproductive age [3], suggesting that most women carry a dual burden of outside labor and family care. Work capacity, therefore, has important financial and social implications for these women.

The dual work burden is made worse by the fact that more than half of Indian women have anemia – and this is especially so in rural areas [4]. Anemia is defined by a low (< 12 g/dL) concentration of hemoglobin (Hb) in the blood [5], which then contributes to a diminished work capacity due to the reduction in the oxygen-transport ability of blood in response to prolonged and heavy exercise (i.e., work). The effects of a diet lacking in sufficient caloric intake, micronutrient, and macronutrient content on anemia and consequent work capacity are well-known [6–9], and recent qualitative research also conducted in Odisha, India, indicates that women tend not to prioritize their own health, relative to the health of their husbands or children [10]. For example, they tend to eat only after everyone else in the family has eaten and thus, are often left with inadequate and poor quality food. Anemia is then exacerbated in these women, as they tend not to seek medical care for their own health and physical weakness is understood to be part of their identity as women [11].

A number of other behavioral factors influence work capacity [8] and physical activity is one such behavior. Insufficient daily physical activity results in poor physical and cardiorespiratory fitness. Workers with lower levels of fitness may be able to perform a similar work output, compared with their fitter counterparts; however, the energy expenditure and physiologic cost of performing that work will be higher [12]. On the other hand, sitting and reclining behaviors may aid in recovery from prolonged work in the heat, thus augmenting subsequent work capacity.

Work capacity can be expressed as maximal aerobic capacity (VO_2max_) [12]. A low VO_2max_ will limit the oxygen-delivery capacity of the cardiovascular system, as well as the oxygen-uptake capacity of the muscle, and these limitations are compounded by anemia. Whereas, VO_2max_ is most precisely measured in the laboratory setting using indirect calorimetry, several field-based tests have been validated that rely on nomograms based on heart rate responses to a standardized sub-maximal exercise test. The Queen’s College Step-Test (QCST) [13] is the simplest of these measures and uses prediction equations to calculate VO_2max_ based on recovery heart rate.

The impact of anemia on work capacity (VO_2max_) has been studied extensively in male and female workers [7]. We know of only one other study that has examined multiple determinants of work capacity simultaneously [8]; however, anemia was not considered in this analysis, there were no objective measures of physical activity, and work capacity was determined by the number of rice bundles produced per hour. Therefore, we used cross-sectional data to examine the multivariable contributions of several sociodemographic, physiologic, and behavioral variables to work capacity (VO_2max_) in women of reproductive age living in rural India. We hypothesize that hemoglobin concentrations and physical activity will be strong independent correlates of predicted VO_2max_, but that these associations would vary by age and/or by level of education.

## Methods

The Reduction in Anemia through Normative Innovations (RANI) project is a norms-based, clustered randomized controlled trial to reduce anemia among women of reproductive age in Odisha, India. The project is being implemented in two blocks (Athamalik and Kishorenagar) in the Angul district of Odisha. Angul was selected for study because it is primarily rural and the prevalence of anemia in that region (44%) is similar to that of Odisha [4]. In the two blocks of Athamalik and Kishorenagar, nearly 25% of people are tribal, 33% are literate, and about half of women work outside of the home [14]. Detailed information on the cluster sampling and randomization methods are described elsewhere [14]. Briefly, 89 clusters of villages were randomized into treatment and control groups on a 1:1 basis by the program implementers, using a random number generator. Women (2055 per group) living in 15 selected clusters (40–41 villages) were then randomly selected for data collection. The overall sample size was based on a predicted 7% improvement in anemia prevalence, assuming an alpha-level of 0.05, with statistical power (1-beta) of 0.80 and accounting for a design effect of 2.0 (clustering effects within the villages) and a 20% loss to follow-up. All women between 15 and 49 years old, who resided in one of the villages selected for study, and who speak Odia were eligible to participate in the larger trial. The sampling design also randomly-generated a subset (187 per group) of non-pregnant participants who performed the QCST and who wore an activity monitor for 3 days. Prior formative research on the cultural norms of this study population indicated that 3 days was the maximal number of days that women would tolerate wearing the ActivPAL. We performed the current cross-sectional analysis using data from this subset of participants, all of whom had complete QCST and physical activity data (*n* = 330). Data collectors and program implementers were blinded with regard to the treatment and control status of the villages. Written informed consent was obtained in Odia by the local data collectors. In the case of participants under the age of 18 years, written permission of one parent or legal guardian and assent of the participants were obtained. All procedures were approved by Institutional Review Boards (IRB) at the George Washington University, as well as Sigma Science and Research, an independent IRB located in New Delhi, India, and the Indian Council for Medical Research’s (ICMR’s) Health Ministry’s Screening Committee (HMSC).

### Sociodemographic and body stature characteristics

Data on age, education (years), caste (scheduled caste, scheduled tribe, other backward caste, other), and parity (number of children) were gathered by questionnaire (Supplemental file). Height and weight were measured on a stadiometer and digital scale, and the body mass index (BMI: weight (kg)/height (m^2^)) was used as an indicator of body stature.

### Work capacity (VO_2max_)

Predicted maximal aerobic capacity (VO_2max_) was determined using a modified QCST [13], which was performed on a step 12 in. in height and at a cadence (determined using a metronome) of 22 steps per minute for 3 min. The height of the step was reduced from 16.25 to 12 in. to accommodate the smaller stature and clothing of the participants. Heart rate (HR) was measured continuously (Polar, Finland) and was recorded while sitting prior to exercise, at 1, 2, 3 min during the test, and at 30- and 60-s of recovery. Predicted VO_2max_ (ml∙(kg∙min)^− 1^) was calculated as [65.81 – (0.1847 x HR (bpm) measured at 30 s of recovery)] [15]. The QCST has demonstrated strong validity against the measurement of the Physical Fitness Index (Pfi) from the Harvard Step Test (r = 0.90; *p* < 0.0001) [16] in women living in India, but may slightly overestimate VO_2max_ compared with a graded exercise test using indirect calorimetry [17, 18]. Percentage of age-predicted maximal heart rate (HR) achieved at 3-min of the test was calculated as [(211–0.64 x age (yrs) / HR at 3-min) × 100] [19] and was used as an additional indicator of work capacity (i.e., energetic efficiency). As cardiorespiratory fitness increases, participants can complete the QCST at a lower percentage of their age-predicted maximal heart rate and, thus, at a lower physiological and energy cost.

### Physical activity, standing, sitting, reclining

Participants were asked to wear an ActivPAL (PAL Technologies, LTD; Glasgow, UK) for three consecutive days to establish baseline measures of daily reclining, sitting, standing, and walking. The ActivPAL is small (53 × 35 × 7 mm), light-weight (15 g) and is attached to the thigh with tegaderm, thereby making it waterproof during bathing. The ActivPAL is capable of recording continuously, and the stored activity profile is retrieved and processed afterward using a personal computer. Thus, participants were blinded from their actual physical activity data during data collection. Data for the different behaviors are expressed as averaged hours/day of sitting, standing, reclining (while awake), and reclining (while sleeping), steps/day or MET-hrs/day. For reference, a metabolic equivalent of task (MET) is the ratio of the metabolic (i.e., energy) cost of a given activity to the resting metabolic rate, and often is used as an indicator of intensity. An activity costing 5 METs (e.g., brisk walking) is performed at 5 times the resting metabolic rate. MET-h/day is a summary measure of physical activity volume throughout the day, and reflects both the intensity and duration of different activities. As this is a rural, agricultural community, there was little day-to-day variation in work activity. Therefore, ActivPAL data were collected on any day of the week, weekdays or weekend days.

### Anemia status

Hemoglobin concentrations (g/dL) were determined from a finger-stick using a HemoCue photometer (HemoCue AB, Angelholm, Sweden). This instrument provides hemoglobin levels immediately and accurately [20]. Anemia was defined as ‘none’ (hemoglobin (Hb) concentrations ≥12 g/dL); ‘mild/moderate’ (Hb > 8 < 12 g/dL) or ‘severe’ (Hb ≤8 g/dL) [5].

### Diet diversity

Dietary quality was assessed by the Food and Agriculture Organization’s Minimum Dietary Diversity for Women (MDD-W) questionnaire during the home interview [21]. For this study, intakes of twenty-two food groups in the previous 24 h were queried by a trained data collector using the list-based method. The MDD-W score was calculated based on 10 food groups including grains, white roots, tubers and plantains, pulses (beans, peas and lentils), nuts and seeds, dairy, meat, poultry and fish, eggs, dark green leafy vegetables, other vitamin A-rich fruits and vegetables, other vegetables, and other fruits. A score of 1 was assigned if women consumed a food belonging to each food group. As recommended [21], women receiving a score of 5 and above were considered to have a diverse diet.

### Statistical analysis

Univariate statistics (mean ± SD and frequencies (%)) first were generated on all study variables. Pearson Product Moment Correlation Coefficients and independent t-tests determined the simple associations between study variables. Predetermined hierarchical (non-multi-level) regression models tested the independent associations between the primary study variables of interest (physical activity, hemoglobin concentrations) and predicted work capacity (VO_2max_) in the presence of age, BMI, education, parity, and diet diversity score. These covariables were chosen based on their established associations with physical activity, hemoglobin concentrations, and/or VO_2max_ in the literature [12, 15, 18, 21–23]. Also, there is evidence that the relationship between age and VO_2max_ is curvilinear [12, 15] and therefore age^2^ was included in the modeling. The first regression model included age, age^2^, BMI, and education. The second model added hemoglobin concentration to the previous model. The third model added physical activity (MET-h/day) to the second model, and the fourth model added in parity. First-order interaction terms were entered into the models individually to determine whether the associations of interest varied by age or level of education. We then repeated this regression modeling while substituting anemia status for the continuous hemoglobin variable. All statistical tests were performed in STATA (v. 16.1) at an α-level of 0.05 (two-sided). The sample size of 330 women allowed more than 80% statistical power (1-β) based on a 16% between-group difference in cognitive performance (the secondary outcome having the greatest amount of error [14]: {[0.50(0.50) + 0.66(0.33)]/(0.50 – 0.66)^2^} ×   7.9 = 149/group [24]. The constant value of 7.9 is derived from the values specified for the α-level (0.05) and 1-β (0.80) according to Whitley and Ball [24].

## Results

Table 1 displays the participant characteristics. On average, women were 30 ± 8 years of age with a BMI of 21.1 ± 3.6 kg/m^2^. Although 18% of the women had no education, approximately 46% had 8 or more years of schooling. The majority of women (59%) were from the “other backward castes” (socially-disadvantaged people that belong to non-Hindu religions) and about 70% had between one and three children. The average hemoglobin concentration was 11.5 ± 1.3 g/dL and the prevalence of anemia in this study population was 61%, which is above the national average of 53% [4]. Of the 201 women having anemia, only four met criteria for severe anemia. Therefore, anemia status was dichotomized into “mild/moderate/severe” and “none” for the regression modeling.

**Table 1:** Descriptive characteristics of the RANI study participants (*n* = 330)

Variable	Mean ± SD	Frequency (%)
Age (y)	30 ± 8	
BMI (kg/m^2^)	21.1 ± 3.6	
Hb concentration (g/dL)	11.5 ± 1.3	
Education (y)		
None		18.4
1–7		35.0
8–13		46.6
Caste		
1 Scheduled caste		13.6
2 Scheduled tribe		24.8
3 Other backward caste		59.5
4 None		2.1
Number of children		
0		23.7
1		18.8
2		36.8
3		14.4
4		5.1
5		1.1
Currently Breastfeeding		
Yes		24.8

Average predicted VO_2max_ in the women was 42.9 ± 3.2 ml∙(kg∙min)^− 1^, indicating an excellent level of cardiorespiratory fitness in women of this age [25]. Participants were able to complete the QCST at about 78% of their age-predicted maximal heart rate; however, the range in physiological cost was 57% (more fit) to 100% (less fit). Overall, women performed an average of about 36 MET-h/day of physical activity and 13,425 steps/day. For reference, 36 MET-h can be achieved by performing work activity of moderate intensity (e.g., 4 METs) for 9 h over the course of the day. Time spent sitting and reclining were low, relative to more affluent and sedentary populations in the United States [26]. Table 2 displays mean levels of predicted VO_2max_ and age-predicted maximal heart rate, as well as the physical activity variables by anemia status. As indicated, there were no significant differences in any of these variables between those women with and without anemia, suggesting that the presence of anemia did not impair work capacity or increase the physiological cost of the QCST. Moreover, we did not observe any behavioral adjustments to anemia, such as lower amounts of overall physical activity or greater amounts of daily sitting and reclining. Data on dietary diversity were available on only 65 women in this subsample of participants. There was no difference in diet diversity score between those with (4.0 ± 0.91) and without (4.3 ± 0.25) anemia.

**Table 2:** Work Capacity and Physical Activity Characteristics by Anemia Status

	Mild, Moderate, and Severe Anemia (Hb = 10.7 ± 1.1 g/dL)	No Anemia (Hb = 12.7 ± 0.6 g/dL)
Predicted VO_2max_ (ml∙(kg∙min^−1^))	42.8 ± 0.27	42.9 ± 0.23
Percent of age-predicted maximal heart rate (%)	78 ± 0.01	77 ± 0.01
MET-h/day	36.1 ± 0.16	36.1 ± 0.14
Steps/day	13,487 ± 383	13,374 ± 332
Sitting (h/day)	6.6 ± 0.17	6.4 ± 0.14
Standing (h/day)	5.4 ± 0.16	5.5 ± 0.14
Reclining (not asleep) (h/day)	0.72 ± 0.10	0.70 ± 0.10

^a^Data are mean ± SE based on 330 women with complete ActivPAL data

The results of the multivariable regression modeling indicated that age^2^, BMI, educational attainment, and physical activity were significant correlates of work capacity in our study participants (Table 3). Predicted VO_2max_ was 0.19 ml∙(kg∙min)^− 1^ lower with each unit increase in BMI (about 2.4 kg in this study population) and was 1.35 ml∙(kg∙min)^− 1^ lower in women with 8–11 years of education compared with those women having no education. Each MET∙h/day increase in physical activity was associated with an increase in predicted VO_2max_ of 0.19 ml∙(kg∙min)^− 1^. Hemoglobin concentration was marginally associated with work capacity in the presence of the other covariables (*p* = 0.067). Predicted VO_2max_ was 0.22 ml∙(kg∙min)^− 1^ higher with each g/dL increase in hemoglobin concentration. When hemoglobin concentration was replaced with anemia status the results were essentially unchanged. Women with anemia had a predicted VO_2max_ 0.23 ml∙(kg∙min)^− 1^ lower than women without anemia (beta = − 0.23; 95% CI: − 0.90, 0.44, although this was not statistically significant (*p* = 0.50). Also, when MET∙h/day was replaced with steps/day as the indicator of physical activity, the estimates also were unchanged. Hours per day spent reclining, sitting, or standing and dietary diversity score were not significantly associated with work capacity in this model. Contrary to our hypothesis, we observed no statistically significant interactions between the study variables of interest and either age or education level in their joint associations with work capacity.

**Table 3:** Step-wise regression estimates describing the multivariable correlates of work capacity (*N* = 330)

	Model 1		Model 2		Model 3		Model 4	
	Estimate	95% CI	Estimate	95% CI	Estimate	95% CI	Estimate	95% CI
Age	0.03	−0.01, 0.08	0.03	−0.02, 0.07	0.02	− 0.03, 0.06	− 0.01	− 0.06, 0.05
Age^2^	**− 0.01**	**− 0.01,-.001**	**−0.01**	**− 0.01,-0.001**	**− 0.01**	**− 0.01,-0.001**	**−0.01**	**− 0.01, 0.00**
BMI	**−0.21**	**− 0.30, − 0.12**	**−0.22**	**− 0.31, − 0.12**	**−0.19**	**− 0.29, − 0.10**	**−0.19**	**− 0.28, − 0.09**
Education								
None	–	–	–	–	–	–	–	–
1–7 y	−0.46	− 1.41, 0.48	−0.52	− 1.46, 0.42	− 0.49	−1.43, 0.45	−0.46	− 1.40, 0.47
8–13 y	**− 1.43**	**− 2.42, − 0.44**	**−1.55**	**− 2.54, − 0.56**	**−1.41**	**− 2.40, − 0.42**	**−1.35**	**−2.34, − 0.36**
Hb (g/dL)			**0.28**	**0.04, 0.52**	0.22	− 0.02, 0.47	0.22	− 0.02, 0.47
MET∙h/day					**0.21**	**0.02, 0.39**	**0.19**	**0.00, 0.38**
Parity							0.27	−0.10, 0.63
	**R**^**2**^ **= 0.13**		**R**^**2**^ **= 0.15**		**R**^**2**^ **= 0.16**		**R**^**2**^ **= 0.16**	

Estimates in **bolded print** are statistically significant at *p* < 0.05

**Fig. 1:**
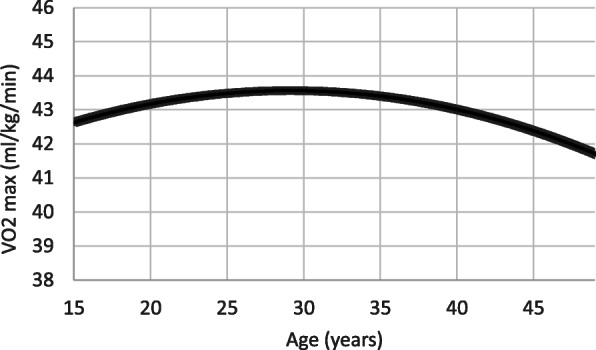
The curvilinear association between age and maximal aerobic capacity (VO_2max_), adjusted for body mass index, education, hemoglobin concentration, physical activity and parity

Fig. 1 shows the inverse curvilinear nature of the adjusted relationship between age and work capacity. Maximal aerobic capacity appeared to increase slowly until reaching its peak value at about age 30 years (approximately the sample mean). Thereafter, VO_2max_ began to decline in an accelerated manner.

## Discussion

Haas and Brownlie [7] present a comprehensive framework to conceptualize the effects of anemia on the physiological, psychosocial, and socioeconomic aspects of work. As stated previously, the underlying mechanism of a diminished work capacity with anemia is a reduction in the oxygen-transport ability of the blood in response to prolonged and heavy exercise. This altered work performance (i.e., capacity, efficiency, and endurance), in turn may affect both the quantity and quality of time allocated to other family responsibilities (cooking and child care) and to leisure activities that allow for recovery from outside work activities. Kalasuramath and colleagues [6] studied 600 working Indian women (18–55 years) and observed that women having mild-to-moderate anemia (Hb > 8 to < 12 g/dL) had a significantly lower VO_2max_ [38.7 ± 6.2 mL∙(kg∙min)^− 1^)], compared with those without anemia [45.5 ± 5.3 mL∙(kg∙min)^− 1^; *p* < 0.05]. In contrast, we observed no difference in predicted VO_2max_ or age-predicted maximal heart rate from the QCST between women with and without anemia is our study population, suggesting that anemia did not impair work capacity or increase the physiological cost of the step-test itself. Moreover, we did not observe any behavioral adjustments to anemia, such as lower amounts of overall daily physical activity or greater amounts of daily sitting and reclining. This is contrary to what we hypothesized and may be explained by the fact that we did not include pregnant women in our study sample and that the mean hemoglobin concentrations between those with and without anemia were very similar. On the other hand, inequitable gender norms may make it very difficult for women to rest during the day if they are feeling fatigued due to anemia. Indeed, these women must bear the burden of both outside labor and family care, and therefore, there is little opportunity (or encouragement) to sit or recline during the day [11].

An average predicted VO_2max_ of about 43 ml∙(kg∙min)^− 1^ indicated an excellent level of cardiorespiratory fitness for women of this age [25]. For comparison, the average VO_2max_ in untrained, but healthy U.S. women of comparable age is typically between 27 and 31 ml∙(kg·min)^− 1^ [25]. Women in the current study achieved 36 MET-h/day and 13,425 steps/day of physical activity, which is well above current WHO guidelines of 150 min/week of moderate-intensity activity (or about 7–8 MET-h/week) [27]. This volume of physical activity in our study sample presumably is due to work activity of moderate intensity (3.5 to 5 METs) that may occur for up to 9 or more hours/day.

We also observed an inverse association between predicted work capacity and level of educational attainment, which is commonly observed in developing countries and presumably due to the fact that more educated women work at more sedentary professions. Other studies of more affluent female university or medical students living in India report predicted VO_2max_ levels between 35 and 38 mL∙(kg∙min)^− 1^ [18, 22], which is lower that what we observed in our rural sample. As is observed in other Indian populations [18, 23] and most populations globally, predicted VO_2max_ was lower with increasing BMI, and this association was independent of the confounding effects of age, parity, and lower levels of physical activity.

Maximal aerobic capacity declines between 5 and 15% per decade after age 25 years [12, 15]. The slightly accelerated decline in VO_2max_ that we observed in our participants after age 30 years has important implications for their health and function. Indeed, if work productivity is expected to remain stable through the reproductive years, then it would be performed at an exponentially higher physiology cost.

Factors other than anemia may affect work capacity in women, and despite the important contributions of age, BMI, education, and physical activity, our final regression model explained only 16% of the variance in predicted VO_2max_. This suggests that other unmeasured behavioral or health-related factors may contribute to predicted VO_2max_ in these women. When the dietary diversity score was added to the regression modeling, the predicted variance of the model decreased further and with no significant contribution from diet. It is possible that total energy intake is a more important correlate of work capacity than is the diversity of one’s diet. Moreover, we did not study iron deficiency (Fe < 15 μg/L), as these measurements are quite expensive to perform in the field. Evidence indicates that the effects of iron deficiency on work performance are partially distinct from those of anemia [7]. Anemia targets oxygen delivery and thus impairs aerobic capacity (which is best captured by the determination of VO_2max_), whereas, iron deficiency affects tissue oxidative capacity (i.e., diffusion of oxygen to the muscle mitochondria for energy production), which is related more to work efficiency and endurance. We attempted to capture endurance by objectively measuring volume (MET∙h/day) of physical activity and by steps/day, while efficiency was assessed by age-predicted maximal heart rate achieved at the conclusion of the QCST; however, we have no way of knowing whether these physical performance outcomes differed between those with and without iron deficiency.

It is important to consider that from an economic standpoint, work *capacity* may differ from actual work *productivity* for many reasons [28]. Work productivity (i.e., output) depends on intelligence, body stature, energy intake, and strength [8], but also on incentives and motivation [29]. Unfortunately, we did not gather information on these latter two factors. Furthermore, not all work requires maximal aerobic capacity; in fact, the rural agricultural, cooking, and family-care labor of our study population may rely more on endurance (i.e., moderate-intensity activity over many hours of the day).

There are several strengths to this study; namely, a sampling design that produced a representative sample of women of reproductive age living in the Angul district and the objective measurement of physical activity and maximal aerobic capacity by validated methods. Nonetheless, the analysis was cross-sectional from baseline RANI data and therefore the temporal sequencing between study factors such as physical activity and hemoglobin concentrations and the primary outcome of VO_2max_ could not be established. Pregnant women were not included in our study sample and perhaps we would have observed stronger associations between physical activity, hemoglobin concentrations, and VO_2max_ if they were included, due to the added physiologic challenge of pregnancy. Also, the difference in mean hemoglobin concentrations between those with and without anemia was only 2 g/dL, which would explain the similarity in regression estimates when using the continuous hemoglobin variable versus the dichotomous anemia variable. Unfortunately, the small number of severe anemia cases (*n* = 4) precluded our ability to study the impact of anemia on work capacity over a range of levels. Finally, due to the small stature of our participants and the fact that they wore a sari while performing the QCST, the height of the step was lowered from 16.25 in. to 12 in.. This adjustment allowed all participants to complete the test; however, it limits our ability to compare our VO_2max_ data with those from other studies using different step heights. In addition, this adjustment may result in an over-estimation of maximal aerobic power in our participants.

## Conclusion

Factors other than anemia may contribute to a lower work capacity in women of reproductive age. Our data indicate that older age, higher BMI and educational attainment, and lower levels of physical activity are important correlates of predicted VO_2max_. Government and non-governmental agencies should account for these factors when creating policies directed toward promoting the economic capacity of women living in rural India.

## Supplementary Information

**Additional file 1.** RANI Baseline Survey

## Data Availability

The datasets used and/or analyzed for the current study are available from the corresponding author on reasonable request.

## Abbreviations

BMI: Body Mass Index
Fe: Blood iron concentration
Hb: Hemoglobin
HR: Heart Rate
MDD-W: Minimum Dietary Diversity for Women
MET: Metabolic Equivalent of Task
Pfi: Physical Fitness Index
QCST: Queen’s College Step Test
RANI: Reduction in Anemia through Normative Interventions
