# Supplementary material for: Determinants of work capacity (predicted VO2max) in non-pregnant women of reproductive age living in rural India
Source: BMC Public Health. 2021 Apr 15;21:735. doi: 10.1186/s12889-021-10785-x (PMC8051129; doi:10.1186/s12889-021-10785-x)
Supplement: Supplementary file 1 — Additional file 1. RANI Baseline Survey [file 12889_2021_10785_MOESM1_ESM.docx]

| **Survey Information** | | | |
| --- | --- | --- | --- |
| Variable Name | **Variable Description** | **Variable Values** | **Freq or M(SD)** |
| block |  | 1 Athmallik  2 Kishorenagar |  |
| cluster |  | provided upon request |  |
| village |  | in datasheet |  |
| HUIC | ID variable |  |  |
| Coordinator | value of coordinator who oversaw survey team |  |  |
| interviewer | value of interviewer who administered survey |  |  |
| first_visit_date | date of first interview |  |  |
| first_visit_time | time of first interview |  |  |
| respondent_present1 | was the respondent present during first visit | 1 present  2 temporarily absent  3 permanently absent |  |
| second_visit_date | date of second interview |  |  |
| second_visit_time | time of second interview |  |  |
| respondent_present2 | was the respondent present during second visit | 1 present  2 temporarily absent  3 permanently absent |  |
| third_visit_date | date of third interview |  |  |
| third_visit_time | time of third interview |  |  |
| respondent_present3 | was the respondent present during third visit | 1 present  2 temporarily absent  3 permanently absent |  |
| consent_respondent | if respondent was present during one of the visits, did they give consent to participate | 0 no  1 written consent  2 verbal consent |  |
| Survey Version | version number |  |  |
| trmt | unblinded treatment arm | 0 control  1 treatment |  |

| Pregnancy Status | | |
| --- | --- | --- |
| Directions: Say, “Today I’m going to ask you about anemia. You may have heard it referred to as ‘lack of blood.’ I will also ask you about iron-folic tablets, also known as IRON BATIKA(tablets). ” Show the participant the IRON BATIKA(tablet) and say, “This is what they look like. Perhaps you have seen them before.”  Say “Before I ask you about anemia and IRON BATIKA, I’d first like to ask you…” | | |
| preg1 | What is your age in years? | Age: __ (years)  999 Unknown |
| preg2 | Are you currently single, married, separated, divorced, Widowed? | 1. Single  2. Married  3. Separated  4. Divorced  5. Widowed |
| preg3 | Are you currently pregnant? | 0. No (skip to preg5)  1. Yes  99. Don’t know (skip to preg5) |
| preg4_week | How many weeks have you been pregnant? | ______ weeks |
| preg4_ month | How many months have you been pregnant? | _______ months |
| preg5 | Are you currently breastfeeding? | 0. No  1. Yes  99. I don’t know  Missing |

| Iron Batika Use | | |
| --- | --- | --- |
| ifa1 | Have you ever eaten/taken an iron batika (tablet)? (Interviewer should hold up the packet of tablets for the interviewee to see) | 0. No, I have never taken it (skip to IFA3)  1. Yes, I am currently taking it  2. Yes, I took in the past, but not currently (skip to IFA3) |
| ifa2 | How many iron batika did you take in the past seven days? | __# of IFA  (If 7 or more, skip to RP1 after) |
| ifa3 | Why did the respondent stop taking IFA? sub questions below | |
| ifa3_1 | stopped IFA because finished tablets | 0. No  1. Yes |
| ifa3_2 | stopped IFA because lost the packet | 0. No  1. Yes |
| ifa3_3 | stopped IFA because experienced dark stool | 0. No  1. Yes |
| ifa3_4 | stopped IFA because experienced nausea | 0. No  1. Yes |
| ifa3_5 | stopped IFA because experienced stomach pain | 0. No  1. Yes |
| ifa3_6 | stopped IFA because fear of big baby | 0. No  1. Yes |
| ifa3_7 | stopped IFA because forgot to take tablets | 0. No  1. Yes |
| ifa3_8 | stopped IFA because did not see any need or benefit | 0. No  1. Yes |
| ifa3_9 | stopped IFA because someone advised to not take or discontinue | 0. No  1. Yes |
| ifa3_10 | stopped IFA because did not like the taste or smell | 0. No  1. Yes |
| ifa3_11 | stopped IFA because gave tablets to someone else | 0. No  1. Yes |
| ifa3_12 | stopped IFA because end of pregnancy | 0. No  1. Yes |
| ifa3_13 | stopped IFA because wasn't anemic anymore | 0. No  1. Yes |
| ifa3_14 | stopped IFA because not in school or school vacation | 0. No  1. Yes |
| ifa3_15 | stopped IFA because I do not need to take the tablets everyday | 0. No  1. Yes |
| ifa3_16 | stopped IFA because no one told me to take it everyday | 0. No  1. Yes |
| ifa3_17 | stopped IFA because did not receive the IFA tablets | 0. No  1. Yes |
| ifa3_88 | stopped IFA because other reason | 0. No  1. Yes |
| ifa4 | Have you asked for iron Batika from anyone for your own use in the last 6 months? | 0. No  1. Yes |
| ifa5_1 | Asked AWW center for IFA | 0. No  1. Yes |
| ifa5_2 | Asked ASHA for IFA | 0. No  1. Yes |
| ifa5_3 | Asked health center for IFA | 0. No  1. Yes |
| ifa5_88 | asked person not on list | 0. No  1. Yes |
| ifa5_other | response to ‘other’ place they asked for IFA from | string variable |

| Risk Perception | | |
| --- | --- | --- |
| Directions: “I am now going to say something. Please tell me if you agree or disagree with it, OK?” Ask participants if they agree with the following statements, or if they are neutral. If they say “agree,” then ask whether they somewhat agree or strongly agree. If they say “disagree”, then ask whether they somewhat disagree or strongly disagree. | | |
| rp1 | Do you think you will become anemic (have lack of blood) in the coming year? | 0. No  1. Yes  2. I am already anemic |
| rp2 | Do you think someone in your family will become anemic (have lack of blood) in the coming year? | 0. No  1. Yes |
| rp3 | If you became anemic (have lack of blood), it would affect your health in a negative way? | 1. Strongly disagree  2. Disagree  3. Neither agree or disagree  4. Agree  5. Strongly agree |
| rp4 | If someone in your family became anemic (have lack of blood), it would affect their health in a negative way? | 1. Strongly disagree  2. Disagree  3. Neither agree or disagree  4. Agree  5. Strongly agree |

| Perceived Access | | |
| --- | --- | --- |
| pa1 | Do you know where you can get iron batika tablets? | 0. No (skip to SE1)  1. Yes |
| pa2 | Do you agree or disagree with the statement, “It is easy for you (or someone) to get iron batika (tablets)?” | 1. Strongly disagree  2. Disagree  3. Neither agree or disagree  4. Agree  5. Strongly agree |

| Self-Efficacy | | |
| --- | --- | --- |
| **Directions: Ask participants if they agree with the following statements, or if they are neutral. If they say “agree,” then ask whether they somewhat agree or strongly agree. If they say “disagree”, then ask whether they somewhat disagree or strongly disagree.** | | |
| se1 | You can take iron batika tablets every week when you are non pregnant. | 1. Strongly disagree  2. Disagree  3. Neither agree or disagree  4. Agree  5. Strongly agree |
| se2 | You believe that you could easily take iron batika | 1. Strongly disagree  2. Disagree  3. Neither agree or disagree 10  4. Agree 1672  5. Strongly agree 1702 |
| se3 | You can take iron batika (tablets) even if your husband/father does not want you to do so | 1. Strongly disagree  2. Disagree  3. Neither agree or disagree  4. Agree  5. Strongly agree  6. Not applicable |
| se4 | You can take iron batika (tablets) even if your mother/mother-in-law does not want you to do so | 1. Strongly disagree  2. Disagree  3. Neither agree or disagree  4. Agree  5. Strongly agree  6. Not applicable |

| Outcome Expectations | | |
| --- | --- | --- |
| Directions: Ask participants if they agree with the following statements, or if they are neutral. If they say “agree,” then ask whether they somewhat agree or strongly agree. If they say “disagree”, then ask whether they somewhat disagree or strongly disagree.  ***NOTE: factor analysis was used to determine that OE scale should be just oe2*oe4 and oe1 and oe3 should be stand alone attitudes*.** | | |
| oe1 | It is important for non pregnant women to take their iron batika (tablets) in one week. | 1. Strongly disagree  2. Disagree  3. Neither agree or disagree  4. Agree  5. Strongly agree |
| oe2 | Taking iron batika (tablets) regularly will make you feel stronger. | 1. Strongly disagree  2. Disagree  3. Neither agree or disagree  4. Agree  5. Strongly agree |
| oe3 | Taking iron batika (tablets) every-day while pregnant will not make the baby big. | 1. Strongly disagree  2. Disagree  3. Neither agree or disagree  4. Agree  5. Strongly agree |
| oe4 | Taking iron batika (tablets) every-day can help prevent fatigue and dizziness during pregnancy. | 1. Strongly disagree  2. Disagree  3. Neither agree or disagree  4. Agree  5. Strongly agree |

| Intentions | | |
| --- | --- | --- |
| Directions: Ask participants if they agree with the following statements, or if they are neutral. If they say “agree,” then ask whether they somewhat agree or strongly agree. If they say “disagree”, then ask whether they somewhat disagree or strongly disagree. | | |
| int1 | If you were to get pregnant in the future, you will take iron batika tablets every-day. | 1. Strongly disagree  2. Disagree  3. Neither agree or disagree  4. Agree  5. Strongly agree |
| int2 | You will take iron batika tablets once a week in the future, even if you are not pregnant. | 1. Strongly disagree  2. Disagree  3. Neither agree or disagree  4. Agree  5. Strongly agree |
| int3 | If you are not pregnant you will take iron batika tablets every week even if your husband/male member in your community does not think it is a good idea. | 1. Strongly disagree  2. Disagree  3. Neither agree or disagree  4. Agree  5. Strongly agree |
| int4 | If you are not pregnant you will take iron batika tablets every week even if your mother-in-law/woman in your community does not think it is a good idea. | 1. Strongly disagree  2. Disagree  3. Neither agree or disagree  4. Agree  5. Strongly agree |

| Social Norms | | | |
| --- | --- | --- | --- |
| I will now ask you your thoughts about what you *think* others in this community (hamlet or village) do. This is not what you think they should do but what you think they actually do. Remember that everything is confidential – we will not share any of your answers and we will not ask you for specific names of people in the community. Also, we are just asking for your best guess to each question. | | | |
| sn1 | What proportion of pregnant women in your community (hamlet or village) take iron batika tablets regularly?  [READ RESPONSE CHOICES ONLY IF NECESSARY] | 0. None  1. Some  2. About half  3. Most  4. All | |
| sn2 | What proportion of Adolescent girl in your community (hamlet or village) take iron batika tablets regularly?  [READ RESPONSE CHOICES ONLY IF NECESSARY] | 0. None  1. Some  2. About half  3. Most  4. All | |
| sn3 | What proportion of non pregnant women in your community (hamlet or village) take iron batika tablets regularly. | 0. None  1. Some  2. About half  3. Most  4. All | |
| I will now ask you your thoughts about what you think others in this community (hamlet or village) *should* do. This is not what you think they actually do, this time I am asking only about what you think they should do. | | | |
| sn4 | How many women in your community (hamlet or village) think you should take iron batika tablets regularly if you are pregnant? | | 0. None  1. Some  2. About half  3. Most  4. All |
| sn5 | How many of the women in your community (hamlet or village) think you should take IRON BATIKA tablets, even when you are not pregnant.  If she is not married, say her mother. | | 0. None  1. Some  2. About half  3. Most  4. All |
| sn6 | For married women :  Your mother-in-law thinks you should take iron batika tablets regularly if you are pregnant.  For unmarried women:  Most mother-in-law think pregnant women should take iron batika tablets regularly.  If she is not married, say her mother | | 1. Strongly disagree  2. Disagree  3. Neither agree or disagree  4. Agree  5. Strongly agree |
| sn7 | For married women :  Your mother-in-law thinks you should take iron batika tablets regularly, even if you are not pregnant.  For unmarried women :  Most mother-in-law think non pregnant women should take iron batika tablets.  If she is not married, say her mother | | 1. Strongly disagree  2. Disagree  3. Neither agree or disagree  4. Agree  5. Strongly agree |
| sn8 | For married women :  Your husband thinks you should take iron batika tablets regularly if you are pregnant  For unmarried women:  Most husband think pregnant women should take iron batika tablets regularly.  If she is not married, say her mother \| | | 1. Strongly disagree  2. Disagree  3. Neither agree or disagree  4. Agree  5. Strongly agree |
| sn9 | For married women Your husband thinks you should take iron batika tablets regularly when you are not pregnant:  For unmarried women Most husband think non pregnant women should take iron batika tablets regularly. | | 1. Strongly disagree  2. Disagree  3. Neither agree or disagree  4. Agree  5. Strongly agree |

| Gender Norms | | | |
| --- | --- | --- | --- |
| “I am now going to ask you some questions about the role of men and women in your community (hamlet or village). There are no right or wrong answers, these are just your opinions, and nobody from this community (hamlet or village) will know how you responded. I will read a statement and then I want you tell me how much you agree or disagree. This first section asks about what you think most families you know are *actually doing*.”  Directions: Ask participants the extent to which they agree or disagree with the following statements. If they say “agree,” then ask whether they somewhat agree or strongly agree. If they say “disagree”, then ask whether they somewhat disagree or strongly disagree  **DESCRIPTIVE NORMS.** | | | |
| **Gender Roles** | | | |
| gn1 | In most families you know, taking care of children is only the woman’s job | | 1. Strongly disagree  2. Disagree  3. Neither agree or disagree  4. Agree  5. Strongly agree |
| gn2 | In most families you know, only men are the ones who earn money for the family | | 1. Strongly disagree  2. Disagree  3. Neither agree or disagree  4. Agree  5. Strongly agree |
| gn3 | In most families you know, boys are more educated than girls | | 1. Strongly disagree  2. Disagree  3. Neither agree or disagree  4. Agree  5. Strongly agree |
| gn4 | In most families you know, there are times when a husband beats (hits) his wife | | 1. Strongly disagree  2. Disagree  3. Neither agree or disagree  4. Agree  5. Strongly agree |
| **Household Power and Control** | | | |
| gn5 | In most families you know, women obey their husbands in all matters | | 1. Strongly disagree  2. Disagree  3. Neither agree or disagree  4. Agree  5. Strongly agree |
| gn6 | In most families you know, only men make decisions about household income and expenses | | 1. Strongly disagree  2. Disagree  3. Neither agree or disagree  4. Agree  5. Strongly agree |
| gn7 | In most families you know, women ask permission from their husbands to get medical treatment of any kind | | 1. Strongly disagree  2. Disagree  3. Neither agree or disagree  4. Agree  5. Strongly agree |
| **Decision Making Power**  Directions**:** remind participants that they are thinking about what they think most families they know actually do. | | | |
| gn8 | In most families you know, husbands make the decision about buying major household items | | 1. Strongly disagree  2. Disagree  3. Neither agree or disagree  4. Agree  5. Strongly agree |
| gn9 | In most families you know, women ask permission from their husband or mother-in-law to leave the house for any reason | | 1. Strongly disagree  2. Disagree  3. Neither agree or disagree  4. Agree  5. Strongly agree |
| gn10 | In most families you know, women stop going to school after they get married. | | 1. Strongly disagree  2. Disagree  3. Neither agree or disagree  4. Agree  5. Strongly agree |
| **Other Oriented** | | | |
| gn11 | In most families you know, women take care of their husbands, children, and in-laws before they take care of themselves | | 1. Strongly disagree  2. Disagree  3. Neither agree or disagree  4. Agree  5. Strongly agree |
| gn12 | In most families you know, women eat last, after all the family members have eaten | | 1. Strongly disagree  2. Disagree  3. Neither agree or disagree  4. Agree  5. Strongly agree |
| gn13 | In most families you know, women eat whatever is left over after the rest of their family has finished eating. | | 1. Strongly disagree  2. Disagree  3. Neither agree or disagree  4. Agree  5. Strongly agree |
| gn14 | In most families you know, women do all of the housework and finish it before taking rest | | 1. Strongly disagree  2. Disagree  3. Neither agree or disagree  4. Agree  5. Strongly agree |
| **Injunctive Norms.**  Directions**:** Say, “This section asks about what most of your people in your community (hamlet or village) believes people should do.” This is not what people are actually doing but what others think they should be doing. For example, maybe in this community people know they *should* wash their hands with soap and water before eating but they don’t do it every time. So now, I’m asking you about what others think should be done, not what they actually do. Is that clear? (Please move ahead only after you are satisfied that they have understood the difference. Remember that there are no right or wrong answers, these are just your opinions, and nobody from this community (hamlet or village) will know how you responded | | | |
| **Gender Roles** | | | |
| gn15 | Most families you know believe that it should only be a woman’s job to take care of the children. | | 1. Strongly disagree  2. Disagree  3. Neither agree or disagree  4. Agree  5. Strongly agree |
| gn16 | Most families you know believe that men should be the only ones who earn money for the family. | | 1. Strongly disagree  2. Disagree  3. Neither agree or disagree  4. Agree  5. Strongly agree |
| gn17 | Most families you know believe that boys *should* be more educated than girls | | 1. Strongly disagree  2. Disagree  3. Neither agree or disagree  4. Agree  5. Strongly agree |
| gn18 | Most families you know believe that women *should* be beaten in certain circumstances. | | 1. Strongly disagree  2. Disagree  3. Neither agree or disagree  4. Agree  5. Strongly agree |
| **Power and Control** | | | |
| gn19 | Most families you know believe that women should obey their husbands in all matters | | 1. Strongly disagree  2. Disagree  3. Neither agree or disagree  4. Agree  5. Strongly agree |
| gn20 | Most families you know believe that only men should be responsible for household income & expenses | | 1. Strongly disagree  2. Disagree  3. Neither agree or disagree  4. Agree  5. Strongly agree |
| gn21 | Most families you know believe that women should ask permission from their husbands to get medical treatment of any kind | | 1. Strongly disagree  2. Disagree  3. Neither agree or disagree  4. Agree  5. Strongly agree |
| **Decision Making Power**  Directions**:** remind participant that they are thinking about what the families they know think they should be doing, not what they are actually doing. | | | |
| gn22 | Most families you know believe that husbands *should* make the decision about buying major household items (e.g., television, refrigerator, bicycle, motor bikes) | | 1. Strongly disagree  2. Disagree  3. Neither agree or disagree  4. Agree  5. Strongly agree |
| gn23 | Most families you know believe women should ask permission from her husband or mother-in-law to leave the house for any reason. | | 1. Strongly disagree  2. Disagree  3. Neither agree or disagree  4. Agree  5. Strongly agree |
| gn24 | Most families you know believe that women should stop going to school after they get married. | | 1. Strongly disagree  2. Disagree  3. Neither agree or disagree  4. Agree  5. Strongly agree |
| **Other Oriented** | | | |
| gn25 | | Most families you know believe that women should take care of their husbands, children, and in-laws before they take care of themselves | 1. Strongly disagree  2. Disagree  3. Neither agree or disagree  4. Agree  5. Strongly agree |
| gn26 | | Most families you know believe that women *should* eat last, after all the family members have eaten | 1. Strongly disagree  2. Disagree  3. Neither agree or disagree  4. Agree  5. Strongly agree |
| gn27 | | Most families you know believe that women should eat whatever is left over after the rest of the family has eaten | 1. Strongly disagree  2. Disagree  3. Neither agree or disagree  4. Agree  5. Strongly agree |
| gn28 | | Most families you know believe that women should do all o the housework and finish it before taking rest | 1. Strongly disagree  2. Disagree  3. Neither agree or disagree  4. Agree  5. Strongly agree |

| Knowledge about IRON BATIKA and other conditions | | |
| --- | --- | --- |
| Directions: Say “I’m going to read you a number of statements. After I finish reading each statement, please tell me if you think it is true or not.” | | |
| know1 | Iron batika prevents anemia (lack of blood) only for young women and it has no effect on older women. | 1. True  2. False  3. Don’t know |
| know2 | Anemia (lack of blood) can be cured by exercising more. | 1. True  2. False  3. Don’t know |
| know3 | Eating dark green leafy vegetables prevents anemia, or lack of blood. | 1. True  2. False  3. Don’t know |
| know4 | Having malaria can make it easier to get anemia or lack of blood. | 1. True  2. False  3. Don’t know |
| know5 | Routine deworming can reduce anemia or lack of blood | 1. True  2. False  3. Don’t know |
| know6 | Anemia can be spread from one person to another through their saliva | 1. True  2. False  3. Don’t know |
| know7 | Eating rice and chapati can prevent anemia. | 1. True  2. False  3. Don’t know |

| Anemia and Health Communication | | |
| --- | --- | --- |
| Directions**:** Ask participants if they agree, disagree, or neither with the following statements. If they say “agree,” then ask whether they somewhat agree or strongly agree. If they say “disagree”, then ask whether they somewhat disagree or strongly disagree. | | |
| comm1 | You often talk to your family about the health of the women in your family. | 1. Strongly disagree  2. Disagree  3. Neither agree or disagree  4. Agree  5. Strongly agree |
| comm2 | You often talk to people in your community (hamlet or village) about the health of the women in your community (hamlet or village). | 1. Strongly disagree  2. Disagree  3. Neither agree or disagree  4. Agree  5. Strongly agree |
| comm3 | You often talk to members of the self-help group about the health of the women in your community. | 1. Strongly disagree  2. Disagree  3. Neither agree or disagree  4. Agree  5. Strongly agree |

| Mental Health | | |
| --- | --- | --- |
| Say “I will now ask you about some of your feelings and thoughts. Please tell me how often you have felt the following in the past week. | | |
| mh1 | You were bothered by things that usually don’t bother you | 1. Rarely or none of the time (less than 1 day)  2. Some or a little of the time (1-2 days)  3. Occasionally or a moderate amount of the time (3-4 days)  4. Most or all of the time (5-7 days) |
| mh2 | Your appetite was poor: you did not feel like eating | 1. Rarely or none of the time (less than 1 day)  2. Some or a little of the time (1-2 days)  3. Occasionally or a moderate amount of the time (3-4 days)  4. Most or all of the time (5-7 days) |
| mh3 | you felt everything you did was difficult | 1. Rarely or none of the time (less than 1 day)  2. Some or a little of the time (1-2 days)  3. Occasionally or a moderate amount of the time (3-4 days)  4. Most or all of the time (5-7 days) |
| mh4 | you felt your behavior was just as good as other people | 1. Rarely or none of the time (less than 1 day)  2. Some or a little of the time (1-2 days)  3. Occasionally or a moderate amount of the time (3-4 days)  4. Most or all of the time (5-7 days) |
| mh5 | You had trouble keeping your mind on what you were doing | 1. Rarely or none of the time (less than 1 day)  2. Some or a little of the time (1-2 days)  3. Occasionally or a moderate amount of the time (3-4 days)  4. Most or all of the time (5-7 days) |
| mh6 | You felt down | 1. Rarely or none of the time (less than 1 day)  2. Some or a little of the time (1-2 days)  3. Occasionally or a moderate amount of the time (3-4 days)  4. Most or all of the time (5-7 days) |
| mh7 | You felt everything you did was difficult | 1. Rarely or none of the time (less than 1 day)  2. Some or a little of the time (1-2 days)  3. Occasionally or a moderate amount of the time (3-4 days)  4. Most or all of the time (5-7 days) |
| mh8 | You felt hopeful about you future | 1. Rarely or none of the time (less than 1 day)  2. Some or a little of the time (1-2 days)  3. Occasionally or a moderate amount of the time (3-4 days)  4. Most or all of the time (5-7 days) |
| mh9 | You thought your life had been a failure | 1. Rarely or none of the time (less than 1 day)  2. Some or a little of the time (1-2 days)  3. Occasionally or a moderate amount of the time (3-4 days)  4. Most or all of the time (5-7 days) |
| mh10 | You felt fearful | 1. Rarely or none of the time (less than 1 day)  2. Some or a little of the time (1-2 days)  3. Occasionally or a moderate amount of the time (3-4 days)  4. Most or all of the time (5-7 days) |
| mh11 | You felt your sleep was restless | 1. Rarely or none of the time (less than 1 day)  2. Some or a little of the time (1-2 days)  3. Occasionally or a moderate amount of the time (3-4 days)  4. Most or all of the time (5-7 days) |
| mh12 | You felt happy | 1. Rarely or none of the time (less than 1 day)  2. Some or a little of the time (1-2 days)  3. Occasionally or a moderate amount of the time (3-4 days)  4. Most or all of the time (5-7 days) |
| mh13 | You talked less than usual | 1. Rarely or none of the time (less than 1 day)  2. Some or a little of the time (1-2 days)  3. Occasionally or a moderate amount of the time (3-4 days)  4. Most or all of the time (5-7 days) |
| mh14 | You felt lonely | 1. Rarely or none of the time (less than 1 day)  2. Some or a little of the time (1-2 days)  3. Occasionally or a moderate amount of the time (3-4 days)  4. Most or all of the time (5-7 days) |
| mh15 | You felt people were unfriendly | 1. Rarely or none of the time (less than 1 day)  2. Some or a little of the time (1-2 days)  3. Occasionally or a moderate amount of the time (3-4 days)  4. Most or all of the time (5-7 days) |
| mh16 | You enjoyed life | 1. Rarely or none of the time (less than 1 day)  2. Some or a little of the time (1-2 days)  3. Occasionally or a moderate amount of the time (3-4 days)  4. Most or all of the time (5-7 days) |
| mh17 | You had crying spells | 1. Rarely or none of the time (less than 1 day)  2. Some or a little of the time (1-2 days)  3. Occasionally or a moderate amount of the time (3-4 days)  4. Most or all of the time (5-7 days) |
| mh18 | You felt sad | 1. Rarely or none of the time (less than 1 day)  2. Some or a little of the time (1-2 days)  3. Occasionally or a moderate amount of the time (3-4 days)  4. Most or all of the time (5-7 days) |

| Functional Health and Well-Being (SF-12) | | |
| --- | --- | --- |
| Directions**:** This survey asks for your views about your health. This information will help keep track of how you feel and how well you are able to do your usual activities.  Thank you for completing this survey!  For each of the following questions, please mark an in the one box that best describes your answer. | | |
| sf1 | How is your health?  READ RESPONSE CHOICES ONLY IF NECESSARY]  () | 1. Excellent  2. Very Good  3. Good  4. Fair  5. Poor |
| Now I'm going to read a list of activities that you might do during a typical day. As I read each item, please tell me if your health now limits you a lot, limits you a little, or does not limit you at all in these activities. | | |
| sf2a | Moderate activities, such as moving or carrying something heavy  READ RESPONSE CHOICES ONLY IF NECESSARY]) | 1. Yes, Limited A Lot  2. Yes  3. No, Not Limited At All |
| sf2b | Climbing several flights of stairs | 1. Yes, Limited A Lot  2. Yes  3. No, Not Limited At All |
| The following two questions ask you about your physical health and your daily activities. | | |
| sf31 | During the past four weeks, how much of the time have you accomplished less than you would like as a result of your physical health? | 1. All of the time  2. Most of the time  3. Some of the time  4. A little of the time  5. None of the time |
| sf3b | During the past four weeks, how much of the time were you limited in the kind of work or other regular daily activities you do as a result of your physical health? | 1. All of the time  2. Most of the time  3. Some of the time  4. A little of the time  5. None of the time |
| The following two questions ask about your emotions and your daily activities. | | |
| sf4a | During the past four weeks, how much of the time have you accomplished less than you would like as a result of any emotional problems, such as feeling depressed or anxious? | 1. All of the time  2. Most of the time  3. Some of the time  4. A little of the time  5. None of the time |
| sf4b | During the past four weeks, how much of the time did you do work or other regular daily activities less carefully than usual as a result of any emotional problems, such as feeling depressed or anxious? | 1. All of the time  2. Most of the time  3. Some of the time  4. A little of the time  5. None of the time |
| sf5 | During the past four weeks, how much did pain interfere with your normal work, including both work outside the home and housework? Did it interfere | 1. Not At All  2. A Little Bit  3. Moderately  4. Quite A Bit  5. Extremely |
| The next questions are about how you feel and how things have been with you during the past four weeks. As I read each statement, please give me the one answer that comes closest to the way you have been feeling; is it all of the time, most of the time, some of the time, a little of the time, or none of the time? | | |
| sf6a | How much of the time during the past four weeks... have you felt calm and peaceful? [READ RESPONSE CHOICES ONLY IF NECESSARY] | 1. All of the time  2. Most of the time  3. Some of the time  4. A little of the time  5. None of the time |
| sf6b | How much of the time during the past four weeks... did you have a lot of energy?  [READ RESPONSE CHOICES ONLY IF NECESSARY] | 1. All of the time  2. Most of the time  3. Some of the time  4. A little of the time  5. None of the time |
| sf6c | How much of the time during the past four weeks... have you felt downhearted and depressed? | 1. All of the time  2. Most of the time  3. Some of the time  4. A little of the time  5. None of the time |
| sf7 | During the past 4 weeks, how much of the time has your physical health or emotional problems interfered with your visiting with friends or relatives? | 1. All of the time  2. Most of the time  3. Some of the time  4. A little of the time  5. None of the time |

| Dietary Diversity N=1026 (only asked in version 4) | | |
| --- | --- | --- |
|  | Was yesterday a special day, like a celebration or feast day or a fast day where you ate special foods or more or less than usual or did not eat because of fasting | 0. No  1. Yes, celebration feast day  2. Yes, fast day |
|  | consumed food from grains yesterday | 0. no  1. yes  98. don’t know |
|  | consumed veggies or roots that are orange-colored in side yesterday | 0. no  1. yes  98. don’t know |
|  | consumed white roots and tubers or plantains yesterday | 0. no  1. yes  98. don’t know |
|  | consumed dark green leafy vegetables yesterday | 0. no  1. yes  98. don’t know |
|  | consumed fruits that are dark yellow or orange inside yesterday | 0. no  1. yes  98. don’t know |
|  | consumed other fruits yesterday | 0. no  1. yes  98. don’t know |
|  | consumed other vegetables yesterday | 0. no  1. yes  98. don’t know |
|  | consumed meat made from animal organs yesterday | 0. no  1. yes  98. don’t know |
|  | consumed other types of meat or poultry yesterday | 0. no  1. yes  98. don’t know |
|  | consumed eggs yesterday | 0. no  1. yes  98. don’t know |
|  | consumed fish or seafood yesterday | 0. no  1. yes  98. don’t know |
|  | consumed beans or peas yesterday | 0. no  1. yes  98. don’t know |
|  | consumed nuts or seeds yesterday | 0. no  1. yes  98. don’t know |
|  | consumed milk or milk product yesterday | 0. no  1. yes  98. don’t know |
|  | consumed insects or other small protein foods yesterday | 0. no  1. yes  98. don’t know |
|  | consumed red palm oil yesterday | 0. no  1. yes  98. don’t know |
|  | consumed oils or fats yesterday | 0. no  1. yes  98. don’t know |
|  | consumed savory and fried snacks yesterday | 0. no  1. yes  98. don’t know |
|  | consumed sweets yesterday | 0. no  1. yes  98. don’t know |
|  | consumed sugar-sweetened beverage yesterday | 0. no  1. yes  98. don’t know |
|  | consumed condiments or seasonings yesterday | 0. no  1. yes  98. don’t know |
|  | consumed any other beverages yesterday | 0. no  1. yes  98. don’t know |

| Cosmic Outcomes | | |
| --- | --- | --- |
| co1 | In the past month, how often have you given money to a beggar? Would you say never, just once or twice, 3 to 5 times, 6 to 10 times, or more than 10 times? | 0. Never  1. Once or twice  2. 3 to 5 times  3. 6 to 10 times  4. More than 10 times |
| co2 | Imagine that someone finds a wallet lying on the road with 5,000 rupees in it. How many people in this community (hamlet or village) would take the money and keep it for themselves? Would you say everyone would keep the money, most would keep the money, about half would keep the money, less than half would keep the money, or no one would keep the money? | 1. Everyone  2. Most  3. About half  4. Less than half  5. No one |
| co3 | In the next 20 years, do you think most people in Odisha will be much happier than they are today, somewhat happier than they are today, about the same as they are today, somewhat sadder than they are today, or much sadder than they are today? | 1. Much happier  2. Somewhat happier  3. About the same  4. Somewhat sadder  5. Much sadder |
| co4 | If your great grandmother were to see India today, do you think she would be extremely happy, somewhat happy, neither happy nor sad, somewhat sad, or very sad? | 1. Extremely happy  2. Somewhat happy  3. Neither happy nor sad  4. Somewhat sad  5. Very sad |

| Violence Against Women | | |
| --- | --- | --- |
| I am now going to ask you about other women in this village who may have been beaten up by their husbands or other men in their lives. | | |
| vam1 | In this village, in your opinion, how many women are beaten by their husbands at least once in 6 months? | 1. None of them  2. Only 1 or 2 of them  3. Many of them  4. Most of them  5. All of them |
| vam2 | If a woman burns the food, do you think it is OK for her husband to beat her? | 0. No  1. Yes |
| vam3 | If a woman is disrespectful to her mother-in-law, do you think it is OK for her husband to beat her? | 0. No  1. Yes |
| vam4 | If a woman does not keep her house neat and clean, do you think it is OK for her husband to beat her? | 0. No  1. Yes |
| vam5 | If you saw a man beating a woman, do you think you would ask the man to stop? | 0. No  1. Yes |
| vam6 | If you saw a man beating a woman, do you think you would distract the man so that he stops beating her? | 0. No  1. Yes |
| vam7 | How strongly do you agree or disagree with the statement that women sometimes deserve to be beaten? | 1. Strongly disagree  2. Disagree  3. Neither agree or disagree  4. Agree  5. Strongly agree |
| vam8 | How strongly do you agree or disagree with the statement that men have a right to discipline their wives by beating them? | 1. Strongly disagree  2. Disagree  3. Neither agree or disagree  4. Agree  5. Strongly agree |

| Demographics | | |
| --- | --- | --- |
| Thank you so much for answering my questions about anemia and IRON BATIKA. I’d like to ask you a few questions about yourself before we finish. | | |
| dem1 | What is your age in years? | ________________ (years) Age  Unknown 999 |
| dem2 | What is the highest level of school you have attended? | 0. None  1. Class-1 (completed)  2. Class-2 (completed)  3. Class-3 (completed)  4. Class-4 (completed)  5. Class-5 (completed)  6. Class-6 (completed)  7. Class-7 (completed)  8. Class-8 (completed)  9. Class-9 (completed)  10. Class-10 (completed)  11. Class-11 (completed)  12. Class-12 (completed)  13. More than Class-12 (completed)  99. No response |
| dem3 | What is your religion? | 1. Hindu  2. Muslim  3. Christian  4. Sikh  5. Buddhist  6. Jain  7. Other |
| dem4 | Are you a part of a caste or tribe? | 0. No  1. Yes  99. I don’t know |
| dem5 | Is this a scheduled caste, a scheduled tribe, other backward class, or none of them? | 1. Scheduled Caste  2. Scheduled Tribe  3. OBC  4. None of them  5. Don’t know |
| dem6 | How many children do you have? | 0. None  1. One  2. Two  3. Three  4. Four  5. Five or more  99. No response |
| dem7 | Do you own a mobile phone? | 0. No  1. Yes (skip to dem11) |
| dem8 | Whose phone do you use most? | 1. Husband/Male Partner  2. Mother  3. Father/Father-in-law  4. Male relative  5. Female relative  6. Friend  7. Children  8. I never use any phone  88. Other |
| dem9 | How often do you have to ask for permission to use mobile phones? | 1. Always  2. Most of the times  3. Rarely  4. Never |
| dem10 | From whom do you need to ask for permission? | 1. Husband/Male Partner  2. Mother  3. Father/Father-in-law  4. Male relative  5. Female relative  6. Friend  7. Children  8. I never use any phone  88. Other |
| dem11 | What is the number of mobile (phone) you use? |  |
| dem12 | Do you share the phone with anyone?  (Only asked if Dem7 is ‘Yes’) | 0. No  1. Yes |
| dem13 | Who do you share most your phone with? | 1. Husband/Male Partner  2. Mother  3. Father/Father-in-law  4. Male relative  5. Female relative  6. Friend  7. Children  8. I never use any phone  88. Other |
| dem14_1 | comfortable making a call on their own | 0. No  1. Yes |
| dem14_2 | comfortable receiving a call | 0. No  1. Yes |
| dem14_3 | comfortable sending an SMS | 0. No  1. Yes |
| dem14_4 | comfortable receiving an SMS | 0. No  1. Yes |
| dem14_5 | comfortable deliberately missing call | 0. No  1. Yes |
| dem14_6 | comfortable using the internet on phone | 0. No  1. Yes |
| dem14_7 | comfortable using WhatsApp | 0. No  1. Yes |
| dem14_8 | comfortable using Facebook on phone | 0. No  1. Yes |
| dem14_9 | not comfortable doing anything on phone | 0. No  1. Yes |
| dem14_88 | comfortable doing "other" on phone | 0. No  1. Yes |
| dem14_other | unlisted item participant feels comfortable doing on their phone | string |
| dem15 | Can you give us the name and phone number of someone who will always know where you are, in case you move in the next two years? |  |
| dem16 | Are you currently a part of a self-help group (SHG)? | 0. No  1. Yes |
| dem17 | Has a doctor, nurse, ANM, ASHA, or healthcare provider ever told you that you have anemia?  ? | 0. No  1. Yes |
| dem18 | Do you currently have anemia or lack of blood? | 0. No  1. Yes  99. I don’t know |
| dem19 | Have you been treated with deworming medication in the past year? | 0. No  1. Yes  99. I don’t know |
| dem20 | Have you had malaria in the past 6 months? | 0. No  1. Yes  99. I don’t know |
| dem21 | Have you had diarrhea in the past 4 weeks? | 0. No  1. Yes  99. I don’t know |
| dem22 | Do you regularly consume tea at the time of or within an hour of eating a meal (like lunch or dinner)? | 0. No  1. Yes |

| Post-Survey questions for interviewer | | |
| --- | --- | --- |
| AnyQuestion | Thank you for taking the time to answer all my questions today. Do you have any questions for me? | Yes/ହଁ ……….…1🡪Remark  No/ନା …………...0 |
| respondent_rating | Please rate how attentive you think the respondent was during this interview | Respondent was very distracted, was not paying attention / ଉତ୍ତରଦାତା ଅନ୍ୟ ମନସ୍କ ଥିଲେ, ଧ୍ୟାନ ଦଉନଥିଲେ-------------------1  Respondent was paying attention some of the time and not paying attention at other times / ଉତ୍ତରଦାତା ବେଳେ ବେଳେ ଧ୍ୟାନ ଦଉଥିଲେ ଏବଂ ଅନ୍ୟ ସମୟରେ ଧ୍ୟାନ ଦଉନଥିଲେ-------------2  Respondent was fully attentive / ଉତ୍ତରଦାତା ସମ୍ପୁର୍ଣ ଧ୍ୟାନ ଦଉଥିଲେ--------------------3 |

ANTHROPOMETERIC AND HB

| **Variable Name** | **Variable Description** | **Variable Values** |
| --- | --- | --- |
|  |  |  |
| AN_Lt | name of lab tech that administered measurements | provided upon request |
| AN_Coordinator | name of coordinator that oversaw measurements | provided upon request |
| AN_date_1 | date of the first visit |  |
| AN_time_2 | time of the first visit |  |
| AN_age | age of the participant |  |
| AN_preg | is the respondent pregnant | 1 pregnant  2 not pregnant |
| AN_child_present | is there a child present during the measurements | 1 child present  2 child not present |
| AN_child_age | what is the age of the child that is present during the measurements |  |
| AN_weight | respondent’s weight in kg |  |
| AN_height | respondent’s height in cm |  |
| AN_Haem_wra | hemoglobin measurement |  |
